# Analysis of risk factors and establishment of early warning model for recent postoperative complications of colorectal cancer

**DOI:** 10.3389/fonc.2024.1411817

**Published:** 2024-11-06

**Authors:** Yang Ou, Yang Yang, Weimin Yang, Yulin Pan, Wu Tian, Zejun Wang, Xianzhe Yu, Jihang Luo, Leibo Wang

**Affiliations:** ^1^ Surgery, Beijing Jishuitan Hospital Guizhou Hospital, Guiyang, China; ^2^ Surgery, Guizhou Orthopaedic Hospital, Guiyang, China; ^3^ Department of Anesthesiology, Affiliated Cancer Hospital of Guizhou Medical University, Guiyang, China; ^4^ Hangzhou Litchi Medical Beauty Clinic, Hangzhou, China; ^5^ Department of Gastrointestinal Surgery, Affiliated Cancer Hospital of Guizhou Medical University, Guiyang, China; ^6^ Department of Gastrointestinal Surgery, Chengdu Second People’s Hospital, Chengdu, China; ^7^ Department of Infection, Affiliated Hospital of Zunyi Medical University, Zunyi, China

**Keywords:** colorectal cancer, postoperative complications, nomogram, PNI, ASA

## Abstract

**Objective:**

This study aimed to analyze factors associated with recent complications after colorectal cancer surgery, constructing a nomogram to aid gastrointestinal surgeons in preoperative decision-making for patients at risk of such complications.

**Methods:**

In this retrospective study, clinical data were collected from patients undergoing radical colorectal cancer surgery at the Department of Gastrointestinal Surgery of the Affiliated Cancer Hospital of Guizhou Medical University and the Second People’s Hospital of Chengdu from November 1, 2021, to January 26, 2024. Univariable and multivariable logistic regression analyses were conducted to assess risk factors for recent postoperative complications and develop a prediction model. External validation was performed using data from 48 postoperative colorectal cancer patients in the Second People’s Hospital of Chengdu City from January 1, 2023, to May 30, 2023. Evaluation included receiver operating characteristic (ROC) curves, calibration curves, and decision curve analysis using R4.2.2 statistical software.

**Results:**

A total of 324 patients who underwent radical colorectal cancer surgery were enrolled. The training cohort (n=176) identified four independent risk factors for recent complications: PNI ≥45 (OR=4.17, P<0.001), Albumin <40 g/L (OR=3.9, P<0.001), ASA score III-IV (OR=6.29, P<0.001), and Tumor diameter ≥5 cm (OR=4.24, P<0.001). A nomogram was constructed incorporating these factors. The AUC of the nomogram model in the training cohort was 0.835, with subsequent internal and external validation cohort AUCs of 0.815 and 0.819, respectively, indicating strong discriminatory ability. The calibration curve demonstrated good consistency, and decision curve analysis indicated high clinical utility.

**Conclusion:**

PNI ≥45, Albumin <40 g/L, ASA score III-IV, and Tumor diameter ≥5 cm emerged as independent risk factors for recent complications following colorectal cancer surgery. We developed a nomogram model for these complications, potentially aiding gastrointestinal surgeons in preoperative patient evaluation and treatment planning for colorectal cancer surgery.

## Introduction

Colorectal cancer (CRC) is one of the most common malignant tumors in the world. According to the latest global data released by the International Agency for Research on Cancer of the World Health Organization, in 2020, there were 1.93 million new cases of colorectal cancer and 935,000 deaths worldwide. It ranks as the third leading cause of cancer incidence and the second leading cause of cancer death (1), posing a significant threat to human life and health. The symptoms of early CRC are insidious, harmful, and difficult to treat, leading to a poor prognosis and a high fatality rate (2). Currently, surgical treatment is the main approach for middle and early CRC (3). In recent years, surgical operations have continuously matured, making the treatment of colorectal cancer relatively perfect, and reducing yearly mortality caused by postoperative complications. However, the incidence of postoperative complications in colorectal cancer patients remains high, ranging from 10% to 37% (4). These complications pose a significant challenge for clinicians, not only increasing patients’ hospital stay and costs, but also significantly impacting surgical efficacy, postoperative rehabilitation, and patient prognosis. In severe cases, postoperative complications can even lead to death. Therefore, understanding the risk factors affecting the postoperative complications of colon cancer patients, and implementing reasonable intervention and control measures for the corresponding risk factors at an early stage, holds important clinical significance.

Existing studies have suggested that factors such as surgical approach, BMI, gender, preoperative anemia, ASA, tumor diameter, TNM stage, and pathological staging are thought to be associated with postoperative complications in colorectal cancer. However, the results of different studies are not completely consistent (5–7). The degree of influence of some risk factors is still unclear, possibly due to differences in demographic characteristics, medical care, and the size of study samples. Currently, there are few reports on the comprehensive risk prediction of postoperative complications in colorectal cancer patients. Additionally, there lacks a clinical model that can accurately predict postoperative complications in colorectal cancer, enabling targeted preventive measures to reduce the risk effectively. Therefore, we aim to focus on variables significantly impacting the risk of postoperative complications. We will explore the validity and applicability of predictive models, assess the risk factors for postoperative complications in colorectal cancer, and develop methods for establishing reliable predictive models. By examining the risk factors and predictive models for postoperative complications, clinicians can access more precise risk assessment tools to aid in making informed treatment decisions.

Therefore, this study conducted a retrospective analysis of case data from patients who underwent radical resection of colorectal cancer at the Affiliated Cancer Hospital of Guizhou Medical University and the Gastrointestinal Surgery Department of Chengdu Second People’s Hospital. The study aimed to identify the risk factors for postoperative complications of colorectal cancer, establish a nomogram prediction model for these complications, and conduct internal and external validation of the developed model. The goal is to assist gastrointestinal surgeons in making preoperative clinical decisions for patients with potential near-term postoperative complications.

## Methods

### Study population and research design

This study employed a retrospective design to analyze the clinical data of 324 patients who underwent radical colorectal cancer surgery at the Department of Gastrointestinal Surgery of the Affiliated Cancer Hospital of Guizhou Medical University from November 1, 2021 to January 26, 2024. The patients’ clinical data were randomly divided into a training cohort and an internal validation cohort in a 7:3 ratio. Additionally, the clinical data of 48 patients who underwent radical surgery for colorectal cancer at the Second People’s Hospital of Chengdu City from January 1, 2023 to May 30, 2023 were used as the external validation cohort. Inclusion Criteria: 1. Patients who underwent surgery for colorectal cancer and were diagnosed with colorectal cancer based on postoperative pathological results. 2. Patients without any distant metastasis. 3. Patients with postoperative TNM clinicopathologic staging of stage I, II, or III. 4. Patients with complete clinical data. Exclusion Criteria: 1. Patients who did not undergo surgical treatment or underwent palliative resection. 2. Patients with stage IV disease. 3. Patients with distant metastases discovered during surgery. 4. Patients who underwent combined organ resection during surgery. 5. Patients with incomplete case data. The institutional ethical review boards of all participating hospitals approved this retrospective study. Due to the low risk associated with retrospective studies, the requirement for informed consent was waived by the ethical review boards.

### Clinical data collection

1. General Baseline Characteristics of Patients: Gender, Age, Body mass index (BMI), Underlying disease (Hypertension, Diabetes, Cardiovascular, COPD, Cerebral infarction), American Society of Anesthesiologists (ASA) classification, History of abdominal surgeries, History of neoadjuvant chemotherapy, Preoperative hemoglobin, Preoperative albumin, Preoperative CEA, Prognostic Nutritional Index (PNI), Neutrophil-to-Lymphocyte Ratio (NLR). 2. Pathological Characteristics: Tumor diameter, Tumor T and N stage, Tumor pathology stage. 3. Characteristics of Surgery-related Information: Surgery approach, Surgery method, Operation time, Intraoperative bleeding. All surgeries were performed by senior attending physicians in the department.

PNI is a nutritional assessment method, defined as PNI = preoperative serum albumin (g/L) + 5 × preoperative lymphocyte count (10^9/L). In this study, it is established that PNI=45 serves as the cut-off value to classify the nutritional state (8, 9). Patients with a PNI <45 are classified in a state of moderately severe malnutrition. Thus, the cut-off value of PNI in this study was 45. Neutrophil/lymphocyte ratio (NLR) is an indicator that reflects the presence of inflammation in the body, commonly utilized in clinical practice to assess its relationship with the development of malignant tumors (10).

### Complication selection

The patient’s medical records, nursing records, consultation records, laboratory tests, and imaging tests were reviewed to identify complications experienced by the patient. Postoperative complications (11): One or more disease-related events that deviate from the normal course of postoperative recovery occur within 30 days from the date of surgery, thereby extending the length of hospital stay. Abdominal and abscess Peritoneal effusion diagnostic criteria (12): fever, body temperature > 38°C, elevated inflammatory markers, positive results of auxiliary examinations such as B-ultrasound or CT, and positive results of microbial culture. Anastomotic leakage: refers to the lack of integrity of the intestinal wall at the anastomosis between the colon and the rectum or the anastomosis between the colon and the anus, which leads to the communication between the inside and outside of the intestinal cavity. Anastomotic leakage was diagnosed when one of the following symptoms was present: (a) persistent abdominal pain, abdominal distension, fever, and peritonitis; (b) Pelvic/abdominal drainage tube, surgical incision or vaginal discharge of gas, purulent or fecal-like fluid; (c) Imaging examination confirmed pelvic abscess near the anastomosis; (d) Contrast agent leaked out from the leakage or from the drainage tube during angiography; (e) Anastomotic defect or dehiscence was confirmed by examination or reoperation (13). Anastomotic hemorrhage, Abdominal hemorrhage, Intestinal obstruction, Wound infection, Lung infection, Pleural effusion, Respiratory failure, Venous thrombosis, Cerebral infarction. Patients were then categorized into two groups: the complication group and the non-complication group, based on the presence or absence of complications.

### Statistical analysis

All statistical analyses in the study were conducted using R 4.2.2 statistical software. Normally distributed measurements were presented as mean ± standard deviation (X ± s), while count data were presented as cases (%). Logistic univariate regression analysis was initially performed on the training cohort of patients’ clinicopathological characteristics. Variables showing statistically significant differences were then included in the multivariate Logistic regression analysis. The independent risk factors identified from the results of the multivariate Logistic regression analysis were used to establish the nomogram model. Internal validation was conducted on this model using the validation cohort. External validation of the nomogram model was performed using an external validation cohort. The discriminatory power of the models was evaluated by calculating the area under the curve (AUC) of the receiver operating characteristic (ROC) curve. An AUC > 0.75 was considered indicative of a good predictive power of the model. Calibration curves were plotted to compare the predicted probability of complications versus the actual probability of complications for the nomogram model, verifying its consistency. Additionally, Decision Curve Analysis (DCA) was utilized to assess the clinical utility and net benefit of the model. Statistical significance was defined as P < 0.05.

## Results

### Incidence of clinically significant postoperative complications

In the cohort of 276 patients from the Affiliated Cancer Hospital of Guizhou Medical University, a total of 62 patients (22.46%) experienced clinically significant complications following surgery. The most common postoperative complications, ranked by frequency, included: Lung infection (12 cases, 4.34%), Wound infection (11 cases, 3.98%), Peritoneal effusion (8 cases, 2.89%), Anastomotic hemorrhage (7 cases, 2.86%; including 244 cases of intestinal anastomosis + protective stoma, colonic anastomosis, Dixon and colorectal anastomosis), Anastomotic leakage (5 cases, 2.24%; including 223 cases of colonic anastomosis, Dixon and colorectal anastomosis), Abdominal abscess (4 cases, 1.44%), Intestinal obstruction (4 cases, 1.44%), Pleural effusion (4 cases, 1.44%), Respiratory failure (3 cases, 1.08%), Abdominal hemorrhage (2 cases, 0.72%), Venous thrombosis (1 case, 0.36%),Cerebral infarction (1 case, 0.36%). In the group of 48 patients from the Second People’s Hospital of Chengdu City, 5 patients (10.41%) experienced clinically significant postoperative complications. The prevalent complications in this group, ranked in order, were: Intestinal obstruction (2 cases, 4.16%), Pulmonary infection (2 cases, 4.16%),Venous thrombosis (1 case, 2.08%), as shown in **Table 1**.

**Table 1:** Postoperative complications in 324 patients with colorectal cancer.

Postoperative complication	Number of cases^a^ (%)	Number of cases^b^ (%)
Abdominal abscess	4 (1.44)	0(0)
Peritoneal effusion	8 (2.89)	0(0)
Anastomotic leakage^C^	5 (2.24)	0(0)
Anastomotic hemorrhage^D^	7 (2.86)	0(0)
Abdominal hemorrhage	2 (0.72)	0(0)
Intestinal obstruction	4 (1.44)	2 (4.16)
Wound infection	11 (3.98)	0(0)
Lung infection	12 (4.34)	2 (4.16)
Pleural effusion	4 (1.44)	0(0)
Respiratory failure	3 (1.08)	0(0)
Venous thrombosis	1 (0.36)	1(2.08)
Cerebral infarction	1 (0.36)	0(0)

Number of cases^a^ (%): Affiliated Cancer Hospital of Guizhou Medical University; Number of cases^b^ (%): Chengdu Second People’s Hospital; C: Anastomotic leakage: postoperative complications after three types of anastomoses: colonic anastomosis, Dixon, and colo-anal anastomosis (256 cases in total); D: Anastomotic hemorrhage: postoperative complications after four types of anastomoses: intestinal anastomosis+protective stoma, colonic anastomosis, Dixon, and colo-anal anastomosis (290 cases in total).

### Clinical data of the patients

A total of 324 clinical data from patients who underwent radical surgery for colorectal cancer at the Affiliated Cancer Hospital of Guizhou Medical University and the Second People’s Hospital of Chengdu were included in this study. The Department of Gastrointestinal Surgery at the Affiliated Cancer Hospital of Guizhou Medical University gathered clinical data from 276 patients, which were then randomly divided into a training cohort (n=196) and an internal validation cohort (n=80) in a 7:3 ratio. In the training cohort, 71 (36.22%) patients were ≥65 years old, 71.94% (n=141) had albumin levels <40 g/L, and 119 (60.71%) experienced a procedure time of 200 minutes or more. For the internal validation cohort, 41 (51.25%) patients had a PNI <45, 38.75% (n=31) had a tumor diameter of 5 cm or more, and 17 (21.25%) had an ASA score of III-IV. In the external validation cohort (n=48), patients ≥65 years old constituted 31.25% (n=15), 54.17% (n=26) of the patients had a procedure time of 200 minutes or more, and 29.17% (n=14) had a tumor diameter of ≥5 cm. A table with the detailed clinical characteristics of the patients is provided in **Table 2**.

**Table 2:** Clinical characteristics of patients in the training, internal, and external validation cohorts.

	Training cohort	Internal validation cohort	External validation cohort	p
N	196	80	48	
Age (%)				0.7325
<65	125 (63.78)	54 (67.50)	33 (68.75)	
≥65	71 (36.22)	26 (32.50)	15 (31.25)	
Gender (%)				0.7467
Male	113 (57.65)	50 (62.50)	29 (60.42)	
Female	83 (42.35)	30 (37.50)	19 (39.58)	
BMI (%)				0.2748
≤18.5	39 (19.90)	16 (20.00)	16 (33.33)	
18.5-25	137 (69.90)	58 (72.50)	27 (56.25)	
≥25	20 (10.20)	6 (7.50)	5 (10.42)	
Anemia (%)				0.2919
YES	60 (30.61)	18 (22.50)	11 (22.92)	
NO	136 (69.39)	62 (77.50)	37 (77.08)	
PNI (%)				0.2653
<45	115 (58.97)	41 (51.25)	23 (47.92)	
≥45	80 (41.03)	39 (48.75)	25 (52.08)	
Albumin (%)				0.3874
<40	141 (71.94)	63 (78.75)	33 (68.75)	
≥40	55 (28.06)	17 (21.25)	15 (31.25)	
Neoadjuvant chemotherapy (%)				0.0295
YES	22 (11.22)	6 (7.50)	11 (22.92)	
NO	174 (88.78)	74 (92.50)	37 (77.08)	
ASA (%)				0.7932
I-II	147 (75.00)	63 (78.75)	37 (77.08)	
III-IV	49 (25.00)	17 (21.25)	11 (22.92)	
Surgical approach (%)				0.529
Laparotomy	11 (5.61)	3 (3.75)	1 (2.08)	
Laparoscope	185 (94.39)	77 (96.25)	47 (97.92)	
Operation time (%)				0.5591
<200	77 (39.29)	29 (36.25)	22 (45.83)	
≥200	119 (60.71)	51 (63.75)	26 (54.17)	
Blood loss (%)				0.9345
<100	134 (68.37)	53 (66.25)	33 (68.75)	
≥100	62 (31.63)	27 (33.75)	15 (31.25)	
Differentiation extent (%)				0.8823
Low	47 (23.98)	24 (30.00)	13 (27.08)	
Medium	134 (68.37)	50 (62.50)	31 (64.58)	
High	15 (7.65)	6 (7.50)	4 (8.33)	
T staging (%)				0.0928
Tis	5 (2.55)	2 (2.50)	2 (4.17)	
T1	9 (4.59)	3 (3.75)	0 (0.00)	
T2	34 (17.35)	9 (11.25)	9 (18.75)	
T3	80 (40.82)	37 (46.25)	30 (62.50)	
T4	68 (34.69)	29 (36.25)	7 (14.58)	
N staging (%)				0.6449
N0	110 (56.12)	43 (53.75)	27 (56.25)	
N1	55 (28.06)	20 (25.00)	10 (20.83)	
N2	31 (15.82)	17 (21.25)	11 (22.92)	
Pathologic stage (%)				0.88
I	39 (19.90)	12 (15.00)	10 (20.83)	
II	65 (33.16)	30 (37.50)	16 (33.33)	
III	92 (46.94)	38 (47.50)	22 (45.83)	
Tumor diameter (%)				0.052
<5	103 (52.55)	49 (61.25)	34 (70.83)	
≥5	93 (47.45)	31 (38.75)	14 (29.17)	
History of abdominal surgery (%)				0.1925
NO	159 (81.12)	70 (87.50)	36 (75.00)	
YES	37 (18.88)	10 (12.50)	12 (25.00)	
Tumor location (%)				0.5807
Rectum	115 (58.67)	51 (63.75)	35 (72.92)	
Sigmoid	19 (9.69)	9 (11.25)	3 (6.25)	
Descending colon	13 (6.63)	7 (8.75)	1 (2.08)	
Transverse colon	6 (3.06)	1 (1.25)	1 (2.08)	
Ascending colon	43 (21.94)	12 (15.00)	8 (16.67)	
Hypertension (%)				0.7577
NO	150 (76.53)	63 (78.75)	39 (81.25)	
YES	46 (23.47)	17 (21.25)	9 (18.75)	
Diabetes (%)				0.229
NO	177 (90.31)	77 (96.25)	43 (89.58)	
YES	19 (9.69)	3 (3.75)	5 (10.42)	
Cardiovascular (%)				0.8681
NO	186 (94.90)	75 (93.75)	46 (95.83)	
YES	10 (5.10)	5 (6.25)	2 (4.17)	
COPD (%)				0.7491
NO	184 (93.88)	76 (95.00)	44 (91.67)	
YES	12 (6.12)	4 (5.00)	4 (8.33)	
Cerebral infarction (%)				0.0694
NO	192 (97.96)	75 (93.75)	48 (100.00)	
YES	4 (2.04)	5 (6.25)	0 (0.00)	
NLR (mean (SD))	3.694 (3.037)	3.198 (1.933)	3.190 (2.209)	0.2651

### Screening of risk factors associated with recent complications after colorectal cancer surgery

The results revealed that the independent risk factors for the development of recent complications after colorectal cancer surgery were PNI, albumin levels, ASA, and tumor diameter (as shown in **Table 3**).

**Table 3:** Univariate and multivariate logistic regression analysis of recent complications after radical resection of colorectal cancer.

Variables	Univariable analysis		Multivariable analysis	
	OR (95% CI) P	P	OR (95% CI) P	P
Age				
<65				
≥65	1.49 (0.66-3.39)	0.34		
Gender				
Male				
Female	0.77 (0.33-1.79)	0.55		
Hypertension	0.71 (0.25-1.99)	0.51		
Diabetes	0.72 (0.16-3.29)	0.67		
Cardiovascular	4.72 (1.24-18.01)	0.02	4.33 (0.76-24.6)	0.0977
COPD	0.55 (0.07-4.46)	0.58		
Cerebral infarction	1.59 (0.17-14.76)	0.68		
History of abdominal surgery				
YES	0.72 (0.23-2.21)	0.56		
NO				
BMI (kg/m²)				
≥25				
18.5-25	0.89 (0.33-2.4)	0.81		
≤18.5	0.61 (0.11-3.35)	0.57		
PNI				
<45	0 (0-lnf)	0.99	0 (0-lnf)	0.9949
≥45	4.17 (1.72-10.08)	<0.001	19.17 (4.5-81.69)	0.0001
NLR	1.04 (0.93-1.18)	0.47		
Neoadjuvant chemotherapy				
YES	0.27 (0.03-2.1)	0.21		
NO				
Anemia				
YES				
NO	1.06 (0.43-2.57)	0.91		
Albumin, (g/L)				
<40	3.9 (1.69-9.01)	<0.001	7.2 (1.91-27.12)	0.0035
≥40				
CEA, (ug/L)				
<5				
≥5	2.03 (0.89-4.62)	0.09		
ASA				
I-II				
III-IV	6.29 (2.66-14.88)	<0.001	3.39 (1.13-10.17)	0.0297
Surgical approach				
laparotomy				
Laparoscope	0.25 (0.07-0.92)	0.04	0.34 (0.07-1.76)	0.2004
Operative method				
Right hemicolectomy				
Left hemicolectomy	1.36 (0.13-14.02)	0.8		
Transverse colectomy	1.09 (0.19-6.06)	0.93		
Rectosigmoidectomy	0.79 (0.3-2.05)	0.63		
Operation time,(min)				
<200				
≥200	2.02 (0.81-5.04)	0.13		
Intraoperative				
Blood loss, (ml)				
≥100	3.24 (1.41-7.44)	0.01	2.6 (0.88-7.67)	0.0823
<100				
Tumor diameter, (cm)				
<5				
≥5	4.24 (1.7-10.59)	<0.001	5.43 (1.63-18.07)	0.0058
T staging				
Tis				
T1	1956420.1 (0-lnf)	0.99		
T2	2086848.11 (0-lnf)	0.99		
T3	2235908.69 (0-lnf)	0.99		
T4	3353863.03 (0-lnf)	0.99		
N staging				
N0				
N1	0.34 (0.09-1.22)	0.1		
N2	2.04 (0.78-5.36)	0.15		
Pathologic stage				
I				
II	1.59 (0.46-5.47)	0.46		
III	1.44 (0.44-4.73)	0.55		
Tumor location				
Rectum				
Sigmoid	0.73 (0.15-3.46)	0.69		
Descending colon	1.12 (0.23-5.55)	0.89		
Transverse colon	1.24 (0.14-11.29)	0.85		
Ascending colon	1 (0.36-2.76)	0.99		
Differentiation extent				
Low				
Medium	0.66 (0.27-1.58)	0.35		
High	0 (0-lnf)	0.99		

### Establishment of the nomogram model for recent postoperative complications of colorectal cancer

**Figure 1:**
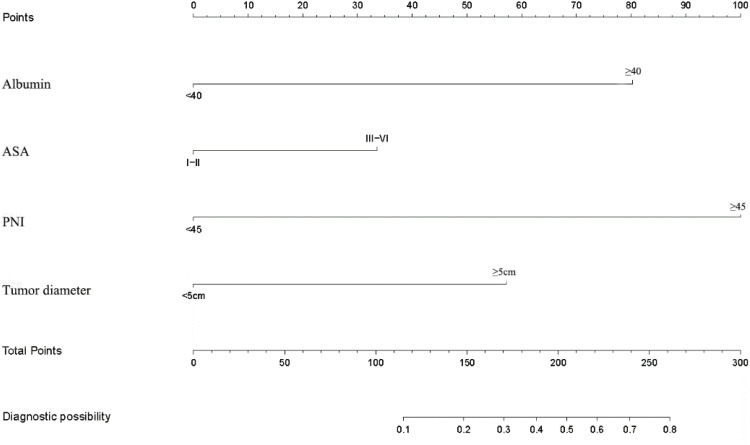
Normogram model of recent postoperative complications of colorectal cancer.

Based on the results of univariate and multivariate logistic regression analysis of the training cohort data, we constructed a nomogram for PNI, albumin levels, ASA, and tumor diameter. The nomogram for postoperative short-term complications indicated that PNI had the greatest impact on postoperative short-term complications (as depicted in **Figure 1**).

### Verification of the prediction model

**Figure 2:**
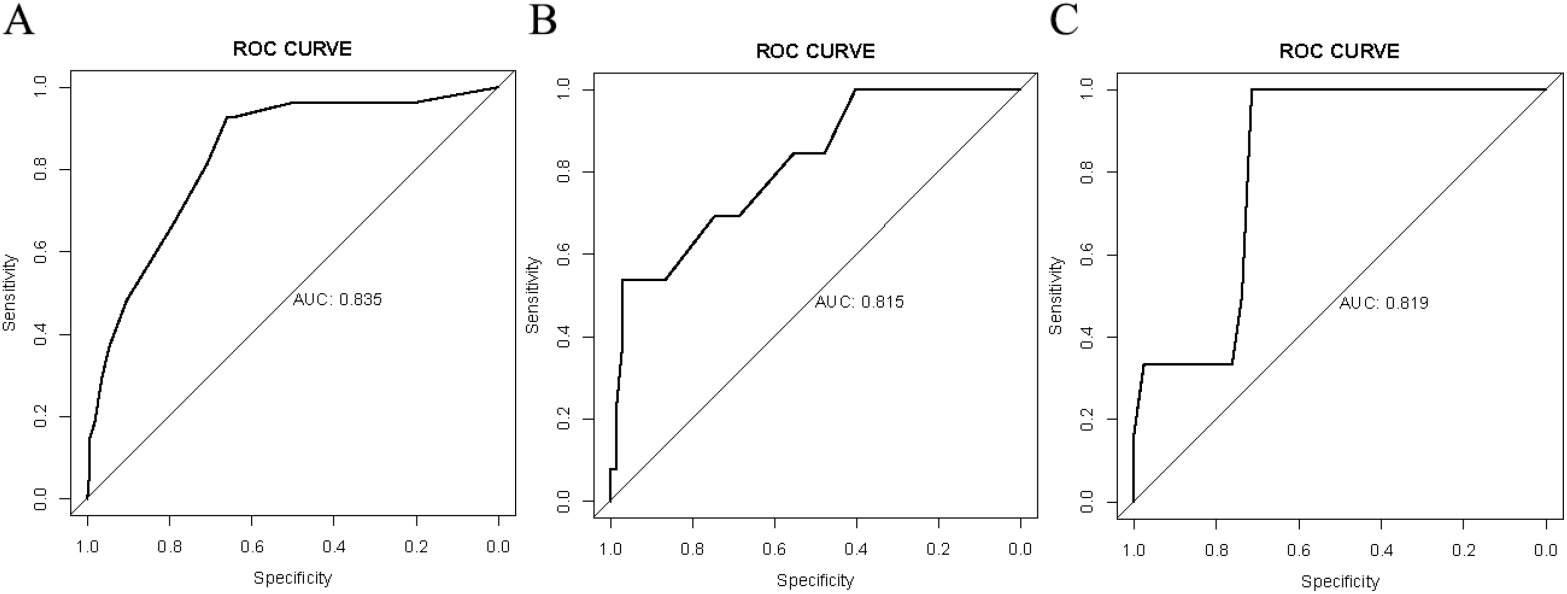
Receiver operating characteristic (ROC) analysis for the nomogram model. **(A)** ROC analysis based on the training cohort. **(B)** ROC analysis based on the internal validation cohort. **(C)** ROC analysis based on the external validation cohort.

**Figure 3:**
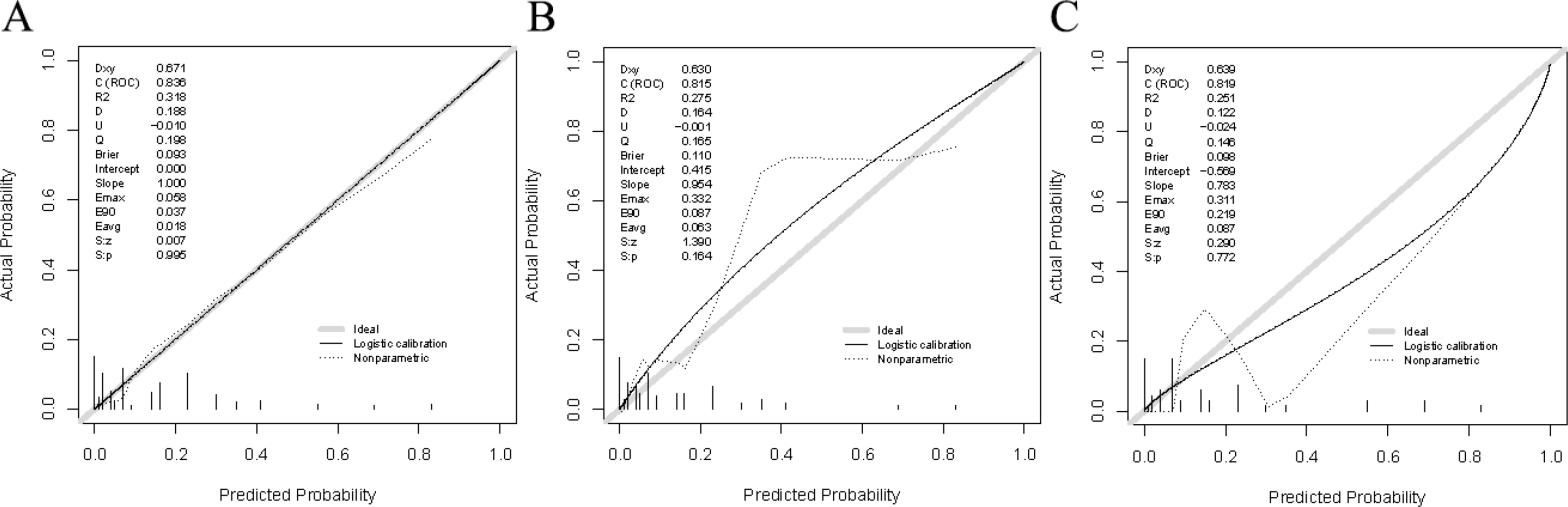
Calibration plots for the nomogram model. **(A)** Calibration plot based on the training cohort. **(B)** Calibration plot based on the internal validation cohort. **(C)** Calibration plot based on the external validation cohort.

**Figure 4:**
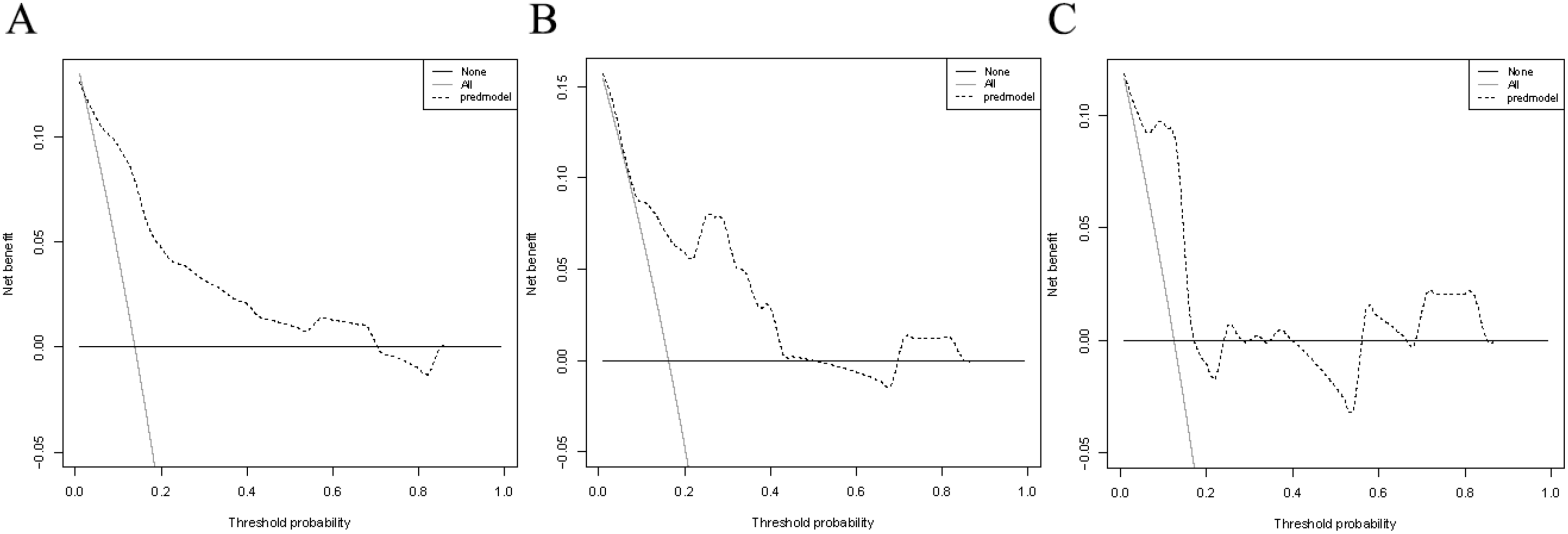
Decision curve analysis for the nomogram model. **(A)** DCA based on the training cohort. **(B)** DCA based on the internal validation cohort. **(C)** DCA based on the external validation cohort.

Verification was conducted in both the internal and external verification cohorts, with the area under the receiver operating characteristic curve (AUC) calculated for quantitative differentiation. The AUC values for the training cohort, internal verification cohort, and external verification cohort were 0.835, 0.815, and 0.819, respectively, as depicted in **Figures 2A–C**. The calibration curve illustrated that the trend of the simulated curve closely matched the actual curve, indicating good consistency for the Nomogram model in this study (**Figures 3A–C**). Additionally, we conducted Decision Curve Analysis (DCA) to assess the clinical utility of the Nomogram model. In most threshold probability ranges, the Nomogram model displayed higher net returns, as demonstrated in **Figures 4A–C**.

## Discussion

Despite advancements in surgical and anastomotic techniques, as well as the utilization of new instruments, gastrointestinal surgeons have made significant progress in performing radical surgery for colorectal cancer. However, postoperative complications remain a substantial concern, greatly impacting patients’ postoperative quality of life, survival time, and perioperative mortality rates (14). These complications increasingly challenge the practices of many surgeons. In this retrospective study, we gathered clinical data from patients in two hospitals to construct and validate a nomogram model for predicting the risk of recent complications following colorectal cancer surgery. Initially, we analyzed the clinical data from the training cohort through univariate and multivariate logistic regression analyses. The results revealed that PNI, albumin levels, ASA, and tumor diameter emerged as independent risk factors for recent complications after colorectal cancer surgery. Subsequently, we developed a nomogram prediction model for these complications. Moreover, we internally and externally validated the model using separate validation cohorts. The nomogram model demonstrated enhanced accuracy, reliability, and clinical utility, as confirmed by ROC analysis, calibration curve analysis, and clinical decision curve analysis.

In this study, PNI was identified as an independent risk factor for the development of recent complications after colorectal cancer surgery, consistent with findings from previous studies (15). Over recent years, PNI has gained prominence as an indicator of a patient’s nutritional and immune status, showing associations with prognosis in various cancers such as breast, lung, colorectal, and gastric cancers (16–19). Tetsuro Tominaga et al. (20) concluded that low preoperative PNI significantly correlated with postoperative complications, advanced tumor status, and poor prognosis in colorectal cancer patients. Similarly, Chen et al. (21) demonstrated PNI as an independent predictor of survival and severe postoperative complications in CRC patients. Additionally, PNI has been linked to tumor aggressiveness and various clinicopathological features, including tumor volume and TNM stage (22). Notably, in a study involving 717 patients with hepatocellular carcinoma following radical hepatectomy, PNI outperformed other indices such as the Controlling Nutritional Status (CONUT) score, Neutrophil-to-Lymphocyte Ratio, Platelet-to-Lymphocyte Ratio (PLR), and the Glasgow Prognostic Score in predicting disease-free survival (DFS) and overall survival (OS), PNI emerged as the sole independent risk factor in this analysis (23). Therefore, heightened preoperative consideration of PNI indicators can serve as a valuable guide for clinical decision-making, particularly in the context of postoperative recovery and management.

The American Society of Anesthesiologists (ASA) score has also been suggested as another risk factor for recent complications after colorectal cancer surgery, a view supported in the study (OR= 3.39, P=0.0297). The ASA scoring system is a class of scales widely used to assess the general health of patients preoperatively (24). Previous studies have shown that ASA scores are strongly associated with the development of postoperative complications, especially after colorectal surgery, such as anastomotic leakage, as well as a higher risk of anesthesia-related complications (24, 25). JH Park et al. (26) showed that in laparoscopic colorectal cancer surgery, the rates of postoperative complications in patients with ASA scores of 1, 2, and 3 were 21.9%, 28.5%, and 38.2%, respectively. Additionally, the mean hospitalization costs for patients with scores of 1, 2, and 3 were $10,769, $11,756, and $13,906, respectively. Further analyses showed that an ASA score of 3 was an independent risk factor for postoperative complications. D Matsubara et al. (27) showed that for elderly colorectal cancer patients with higher ASA scores, laparoscopic surgery may be more beneficial than open surgery in minimizing postoperative complications, and there was no significant difference in the rate of postoperative complications in these patients compared with those with lower ASA scores. Thus, the risk of near-term complications after colorectal cancer surgery is strongly associated with patients’ ASA scores. These findings emphasize the importance of a thorough evaluation of patients before surgery to better predict complication risk and to inform clinical decision-making. Closer monitoring and more careful postoperative management may be required for patients with higher ASA scores to reduce the incidence of complications and improve the outcome of surgical treatment.

Preoperative hypoalbuminemia is another significant factor affecting postoperative complications of colorectal cancer (OR=3.9, P<0.001). The tumor disease itself consumes more protein, placing patients in a long-term state of high metabolic demand. Surgical trauma also increases patients’ protein and energy consumption, elevating the risk of nutritional deficiency and hypoalbuminemia. Consequently, hypoalbuminemia is not uncommon in colorectal cancer patients, with the percentage of perioperative hypoalbuminemia ranging from about 10% to 57% (28). A Decreased serum albumin concentration usually reflects malnutrition in cancer patients (29), predictive of a poor cancer prognosis. Previous studies have consistently demonstrated that preoperative hypoalbuminemia correlates with postoperative complications, mortality, and overall survival (30–32). Hu WH et al (33). concluded that hypoalbuminemia was significantly associated with deep vein thrombosis, pulmonary embolism, superficial and deep surgical site infections, pneumonia, and infectious shock. Additionally, Hardt J et al. (34) showed that hypoalbuminemia is an independent risk factor for postoperative complications in colorectal cancer. Therefore, paying close attention to the presence of hypoalbuminemia preoperatively and actively correcting it may help reduce the incidence of postoperative complications associated with colorectal cancer and improve the safety of surgery.

In this study, a tumor diameter of ≥5 cm was considered an independent risk factor for the development of recent postoperative complications in colorectal cancer. Several current studies have consistently shown that tumor size is significantly associated with the incidence of postoperative complications in colorectal cancer (35–37). In a multicenter study on independent risk factors for postoperative complications in colorectal cancer, Yasui M et al (38). found that tumor size (≥4 cm) was an independent risk factor for such complications. Patients with larger tumor size were more likely to exhibit adverse features such as poor differentiation, preoperative intestinal obstruction, mucinous subtypes, higher T4 stage, and increased lymph node infiltration compared to those with smaller tumors. However, the predictive value of tumor size in postoperative complications remains controversial (39). This controversy arises from the fact that intestinal malignant tumors exhibit both vertical and horizontal growth directions. Horizontal growth is typically represented by tumor size, whereas the depth of vertical infiltration is reflected by the T stage. The T stage is an important predictive index that cannot be ignored when considering postoperative complications of malignant tumors. Therefore, while tumor diameter is widely recognized as an independent risk factor for postoperative complications of colorectal cancer, further research may be necessary to incorporate the role of both horizontal and vertical tumor growth patterns in predicting these complications.

Nomograms are widely used in medicine as a means of prediction (40). In this study, we constructed a nomogram model for predicting the risk of recent complications after colorectal cancer surgery. The nomogram integrated four independent risk factors: PNI, Albumin, ASA, and Tumor diameter. The nomogram exhibited strong predictive ability in the training cohort, internal validation cohort, and external validation cohort (AUC: 0.835 vs 0.815 vs 0.819), and calibration graphs and decision curve analysis also showed favorable results. Therefore, this study has developed and validated a more accurate prediction tool.

Despite the two hospitals providing reliable data support for this study, several limitations should be acknowledged. Firstly, being a retrospective study, it inevitably introduces selection bias. Secondly, although external validation of the model was conducted, the sample size for external validation was small, potentially impacting the accuracy of postoperative complication prediction. Thirdly, due to limitations in medical conditions, important indices such as preoperative pulmonary function tests, nutritional scores, thrombus scores, other preoperative comorbidities, and the tumor distance from the anus were not included in this study. Fourthly, the study combined recent complications of colon and rectal cancers, possibly increasing heterogeneity due to differing surgical methods, which might result in decreased predictive accuracy of the model. Future prospective multicenter studies are still needed to comprehensively evaluate the risk factors for recent complications following surgery for colorectal cancer.

## Conclusion

In summary, the findings of this study highlight that PNI, Albumin, ASA, and Tumor diameter serve as independent predictors of recent postoperative complications in colorectal cancer patients. The construction of a nomogram model for these complications offers promising application potential for gastrointestinal surgeons, aiding in the preoperative evaluation and formulation of treatment strategies for individuals undergoing colorectal cancer surgery.

## Data Availability

The raw data supporting the conclusions of this article will be made available by the authors, without undue reservation.
